# The Perirhinal Cortex Engages in Area and Layer-Specific Encoding of Item Dimensions

**DOI:** 10.3389/fnbeh.2021.744669

**Published:** 2022-01-04

**Authors:** Nithya Sethumadhavan, Christina Strauch, Thu-Huong Hoang, Denise Manahan-Vaughan

**Affiliations:** ^1^Medical Faculty, Department of Neurophysiology, Ruhr University Bochum, Bochum, Germany; ^2^International Graduate School of Neuroscience, Ruhr University Bochum, Bochum, Germany

**Keywords:** perirhinal cortex, fluorescence *in situ* hybridization, immediate early gene, visual information processing, deep layers, superficial layers, item encoding

## Abstract

The perirhinal cortex (PRC), subdivided into areas 35 and 36, belongs to the parahippocampal regions that provide polysensory input to the hippocampus. Efferent and afferent connections along its rostro-caudal axis, and of areas 35 and 36, are extremely diverse. Correspondingly functional tasks in which the PRC participates are manifold. The PRC engages, for example, in sensory information processing, object recognition, and attentional processes. It was previously reported that layer II of the caudal area 35 may be critically involved in the encoding of large-scale objects. In the present study we aimed to disambiguate the roles of the different PRC layers, along with areas 35 and 36, and the rostro-caudal compartments of the PRC, in processing information about objects of different dimensions. Here, we compared effects on information encoding triggered by learning about subtle and discretely visible (microscale) object information and overt, highly visible landmark (macroscale) information. To this end, nuclear expression of the immediate early gene Arc was evaluated using fluorescence in situ hybridization. Increased nuclear Arc expression occurred in layers III and V-VI of the middle and caudal parts of area 35 in response to both novel microscale and macroscale object exposure. By contrast, a significant increase in Arc expression occurred in area 36 only in response to microscale objects. These results indicate that area 36 is specifically involved in the encoding of small and less prominently visible items. In contrast, area 35 engages globally (layer III to VI) in the encoding of object information independent of item dimensions.

## Introduction

The perirhinal cortex (PRC) is located along the rhinal sulcus and can be divided anatomically into Brodmann areas 35 and 36 (Brodmann, 1909). It consists of six cortical layers, whereby layer IV of area 36 contains only a few granule cells and area 35 is considered to be an agranular cortex (Burwell et al., 1995; Burwell, 2001; Kealy and Commins, 2011).

The PRC has been reported to support the integration of multisensory information, emotional aspects of learning such as fear conditioning, spatial memory, and also perception (for review: Kealy and Commins, 2011). The role of the PRC in object recognition memory is undeniable: over the last decades many studies, using different behavioral approaches alone or in combination with PRC lesions, single unit recordings, or immediate early gene (IEG) analysis, support its involvement in novelty detection, as well as the encoding, consolidation, and retrieval of object recognition memory (Mumby and Pinel, 1994; Zhu et al., 1995a; Aggleton and Brown, 2005; Albasser et al., 2015).

Sensory information reaches areas 35 and 36 of the PRC in a differential manner, e.g., inputs from polymodal associational areas are, in general, very strong, but they target area 36 more heavily, whereas unimodal associational areas project to a higher degree to area 35 (Burwell and Amaral, 1998a). As an essential part of the parahippocampal formation, the PRC serves as a transition area between neocortical areas and the hippocampus that interfaces indirectly, *via* the entorhinal cortex, but also directly transmits information to the hippocampus (for review: Kealy and Commins, 2011). PRC projections originate in most anatomical layers and project stronger to the lateral entorhinal cortex (LEC) than to the medial entorhinal cortex. Weak back projections, traveling mostly to the rostral PRC, arise from layers III and V of mostly the LEC (Burwell and Amaral, 1998a, b). Direct projections to the hippocampus are sparse and are presumed to originate only in superficial PRC layers (Furtak et al., 2007; Agster and Burwell, 2013). In contrast, projections from the hippocampus (stronger from the ventral than the dorsal aspect) target deep layers of preferentially area 35 and to a smaller degree area 36 (Agster and Burwell, 2013). These direct connections, together with the indirect connections to the hippocampus lead one to suspect that a layer-specific engagement in object recognition memory may occur in areas 35 and 36. In line with this possibility, a layer-specific distribution of the different types of glutamatergic receptors has been described for neocortical regions, including the PRC, suggesting layer-specific encoding of e.g., object information (Ziakopoulos et al., 1999; Winters and Bussey, 2005; Palomero-Gallagher and Zilles, 2015).

Anatomical and functional differences along the rostro-caudal axis have also been described (Burwell and Amaral, 1998b; Otto et al., 2000; Burwell, 2001; Furtak et al., 2007; Agster and Burwell, 2009; Albasser et al., 2013; Sethumadhavan et al., 2020). Novel object exposure has been reported to trigger a change in immediate early gene (IEG) expression in caudal areas 35 and 36 (Zhu et al., 1995b; Albasser et al., 2010). Object recognition also triggers gene encoding in these PRC areas: Burke and colleagues examined IEG expression in layer V, or neuronal activity in layer II/III and V of the areas 35 and 36 (Burke et al., 2012a, b), and demonstrated that novel and familiar exploration of objects results in enhanced Arc mRNA expression in layer V (Burke et al., 2012a). In our previous work, we reported that layer II of the caudal area 35, along with the postrhinal cortex (POR), engages in the encoding of information about large objects (Sethumadhavan et al., 2020). Given that the PRC strongly projects to the entorhinal cortex that in turn projects to the hippocampus (Burwell and Amaral, 1998b; Agster and Burwell, 2013; Doan et al., 2019), it is not unlikely that object dimensions also result in layer-specific neuronal encoding within the PRC. This aspect is as yet unclarified. However, it has been reported that novel learning about spatial configurations of large and overt (macroscale) or small and discrete (microscale) objects enables both subregion-specific long-term depression (LTD) and nuclear gene encoding in specific neuronal subcompartments of the hippocampus (Manahan-Vaughan and Braunewell, 1999; Kemp and Manahan-Vaughan, 2008; Hagena and Manahan-Vaughan, 2011; Hoang et al., 2018, 2021).

In the current study, we scrutinized the influence of microscale and macroscale item learning on immediate early gene expression in the PRC to specify the contribution of the layers of areas 35 and 36 to these specific forms of learning. To do this, we analyzed nuclear IEG expression in layers II, III, and (I)V-VI of the middle and caudal areas 35 and 36 that was triggered by novel microscale or macroscale item learning. Our results reveal that layers III and V-VI of area 35 engage in the encoding of both micro- and macroscale items, whereas layer III of area 36 is specifically involved in microscale item encoding.

## Materials and Methods

Seven-to-nine-week-old male Wistar rats were used for this study. Animals had *ad libitum* access to water and food and were housed in temperature (22 ± 2°C) and humidity (55 ± 5%) controlled containers (Scantainer, Scanbur Technology A/S, Karlslunde, Denmark) on a 12 h light/12 h dark cycle (lights on from 7 a.m. to 7 p.m.). All experiments were approved in advance by the animal ethics authority of the Federal government of the state of North Rhine-Westphalia (NRW; Landesamt für Arbeitsschutz, Naturschutz, Umweltschutz und Verbraucherschutz, NRW) and carried out according to the European Communities Council directive of 22 September 2010 (2010/63/EU) for the care of laboratory animals. All efforts were made to reduce the number of rats used.

### Behavioral Experiments

The behavioral paradigms were previously established by our group (Manahan-Vaughan and Braunewell, 1999; Kemp and Manahan-Vaughan, 2004, 2008; Hoang et al., 2018). For this study, three groups of animals were examined: one control group and two test groups (microscale and macroscale paradigms). All animals were handled, habituated to the experimental chamber (40 cm width × 40 cm length × 50 cm height, translucent and removable front wall) for 1 h on two consecutive days and on the following test day.

Control group: On the test day, the brains of the animals of the control group were extracted after the end of the habituation phase in the experimental chamber.

Test groups: On the test day, after the end of the habituation to the experimental chamber, animals spent 5 min exploring a holeboard in which small objects were placed inside the holeboard holes (microscale items, micro), or exploring large landmark objects (macroscale items, macro) that were placed on the floor of the chamber. During exploration, the behavior of the animals was video-taped and later analyzed. Animals that did not explore all objects, or did not explore all holes of the holeboard, were excluded from further analysis and from the study as a whole.

**Figure 1:**
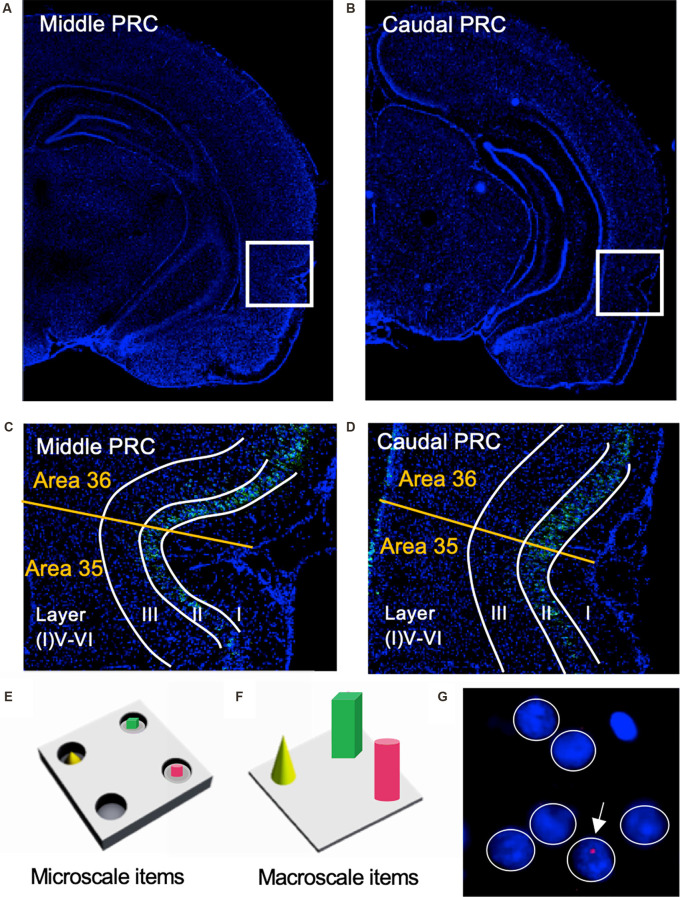
Representation of perirhinal cortex areas and micro- and macroscale item paradigms. **(A,B)** DAPI (4′,6-diamidino-2-phenylindole) stained (blue), nuclei in coronal sections of the rat brain, including outlines highlighting the middle **(A)** and caudal **(B)** compartments of the perirhinal cortex (PRC; indicated by white squares). **(C,D)** Layer organization of the PRC (indicated by white lines). Images were obtained and analyzed in layer II, III, and V-VI/IV-VI of areas 35 and 36 (separated by a yellow line) of the middle **(C)** and caudal **(D)** PRC. To simplify the differentiation of the superficial layers, WFS1 (Wolframin, green) was visualized in some sections of the middle and caudal PRC. Nuclei were stained with DAPI (blue). **(E,F)** Schema of microscale and macroscale item paradigm. Animals participated in a 5 min exploration task, in which small novel objects (microscale items) were placed within three of four holes of a hole board **(E)**, or three large novel objects (macroscale items) were placed on the floor of the chamber **(F)**. **(G)** Example of manual cell counting to identify somatic Arc mRNA FISH expression in the PRC. Nuclei were identified in an experimenter-blind manner and outlined (white circles). Red signals correspond to Arc mRNA expression. Nuclei that contained an Arc mRNA signal within the DAPI stained area were registered as positively labeled nuclei (indicated by white arrow). Nuclei of glial cells, which are characteristically small and strongly stained with DAPI, were excluded from analysis (top, right).

Microscale items: The holeboard (39.8 cm width × 39.8 cm length × 5 cm height, gray) contained four holes (5.5 cm diameter × 5 cm depth), each positioned close to each edge of the holeboard. This holeboard was quickly inserted into the experimental chamber after the habituation phase on the test day. Whereas one hole of the holeboard was left empty, in each of the other three holes, a small item (ca. 2 cm width × 2 cm length × 4 cm height) was placed. These items could only be seen by the animals if they poked their noses into a holeboard hole (Figure 1E). The assignment of the items within the holes of the holeboard was randomly chosen for each animal.

Macroscale items: Three large landmark objects were placed on the floor of the experimental chamber after the habituation phase on the test day. The dimensions of the macroscale items differed: object 1: 10 cm width × 8 cm length × 7 cm height, object 2: 6 cm diameter × 11 cm height, and object 3: 8 cm diameter × 10 cm height (Figure 1F). Each of the items was positioned in one quadrant of the experimental chamber, the assignment of a respective object to a quadrant was randomly chosen for each animal.

The behavioral assessment was conducted as previously described (Hoang et al., 2018; Sethumadhavan et al., 2020). Here, the total exploration time of the animals that was spent in active exploration of the experimental chamber during the 5-min-task was examined (i.e., all behaviors excluding sleeping, grooming, and resting). The time the animals spent exploring each individual object was also assessed.

Activity-dependent expression of nuclear Arc mRNA reaches a peak level 5–6 min after the start of a learning event (Guzowski et al., 1999). We sacrificed the animals and rapidly removed each brain 5–6 min after the commencement of the abovementioned tasks. The brains were directly shock-frozen in 2-methyl butane at −80°C to halt IEG expression at the timepoint of brain extraction.

### Fluorescence *In situ* Hybridization (FISH)

Coronal sections (20 μm thick) containing the PRC were prepared using a Cryostat (Leica CM 3050S), and then directly mounted on glass slides stored at −80°C. For this study areas 35 and 36 of the middle (at ca. −4.56 mm posterior to Bregma), and caudal PRC (at ca. −5.52 mm posterior to Bregma (Paxinos and Watson, 2005) were analyzed (Figures 1A,B). FISH for digoxigenin-labeled Arc was performed as previously described (adapted from Guzowski et al., 1999; Sethumadhavan et al., 2020).

Plasmid containing a full-length cDNA (~3 kb) of the *Arc* transcript (according to Lyford et al., 1995 Genbank: NM_19361.2) Genbank: NM_19361.2) was prepared by Genscript (Genscript Biotech, USA). After linearization and purification steps, digoxigenin-labeled Arc RNA probe was generated using a transcription kit (Ambion^®^ MAXIscript^TM^ T7 Kit, Invitrogen, USA) and RNA labeling mix containing digoxigenin-11-UTP (Roche Diagnostics, Mannheim, Germany). The yield and integrity of the purified RNA probes were verified using gel electrophoresis (agarose 1%).

Slides were fixed in ice-cold 4% paraformaldehyde for 10 min, washed in 2-fold concentrated saline sodium citrate buffer (2× SSC) for 2 min, incubated in acetic anhydride solution for 10 min, and washed three times (1 min, each) in 2× SSC. Then, slides were incubated for 30 min with prehybridization buffer (P1415, Sigma–Aldrich, St. Louis, MO, USA) at room temperature (RT) and hybridized overnight at 56°C with digoxigenin-labeled RNA probes (1 ng/μl in hybridization buffer; H7140, Sigma–Aldrich, St. Louis, MO, USA). Afterward, stringent washing steps were performed as follows: thrice in 2× SSC at 56°C (5 min, each), 15 min in 2× SSC containing RNase A (1 μg/ml) at 37°C, 10 min in 2× SSC at 37°C, 10 min in 0.5× SSC at 56°C, 30 min in 0.5× SSC at 56°C, 10 min in 0.5× SSC at RT, twice in 1× SSC at RT (5 min, each) and thrice in tris-buffered saline (TBS) at RT (5 min, each). The endogenous peroxidase was blocked by hydrogen peroxide treatment for 15 min. After washing in TBS, slides were incubated in animal free blocker (AFB, 1:5, Vectorlabs, USA) and streptavidin (1:5, Vectorlabs, Burlingame, CA, USA) in TBS-Tween for 70 min at RT. Arc-digoxigenin was detected by means of anti-digoxigenin-peroxidase-Fab-fragment (1:2,000, Roche Holding AG, Basel, Switzerland) in AFB (1:5) and biotin (1:5, Vectorlabs, USA) in TBS-Tween. After washing in TBS (thrice for 5 min), the signal was enhanced using biotinylated tyramine in TBS containing H_2_O_2_ for 20 min. Then the slides were washed again in TBS (three times for 5 min). The Arc mRNA signal was visualized by incubating with Streptavidin Cy5 (1:2,000, Dianova, Hamburg, Germany) and AFB (1:5) in TBS-Tween for 90 min at RT. Then slides were washed again in TBS (thrice for 5 min).

### Immunohistochemistry

For a better differentiation of the superficial layers of the PRC we visualized WFS1 (Wolframin, Wolfram syndrome protein) within cells (Takeda et al., 2001; Luuk et al., 2008). For this, after the *in situ* hybridization procedure, single slides underwent the following steps. First, slides were blocked with AFB (1:5) in TBS-Tween for 90 min. Then, WFS1 rabbit polyclonal antibody (1:1,000, 11558-1-AP, Proteintech, Rosemont, IL, USA) diluted in AFB in TBS-Tween was applied on each slide overnight. After washing in TBS, WFS1-labeled cells were visualized by goat anti rabbit-antibody conjugated to Cy2 (Dianova, Hamburg, Germany), and slides were washed again in TBS.

### Sudan Black Staining

After rinsing in TBS, distilled water, and 70% ethanol, the sections were stained using 1% alcoholic Sudan black B (Sigma–Aldrich, St. Louis, MO, USA; Oliveira et al., 2010). Nuclei were visualized using 4′,6-diamidino-2-phenylindole (DAPI) in mounting medium (SCR-038448, Dianova, Hamburg, Germany).

### Image Acquisition

Nuclear Arc mRNA expression was examined in the superficial (layers II and III) and deep (IV-VI) layers of areas 35 and 36 of the middle and caudal PRC (Figures 1C,D). Fluorescent images from the sections were obtained using a slide scanner microscope (20×, Axio Scan.Z1, Zeiss). This approach gave us the opportunity to image a broad area for each of our regions of interest (ROIs) and to analyze a relatively high number of nuclei for each section. For each ROI of each animal, three images from three consecutive slices were obtained and analyzed.

### Data Analysis

We manually quantified the relative expression of nuclear Arc mRNA in pyramidal and non-pyramidal cells of each region of interest (ROI) in the PRC (Figure 1G, white circles). To do so, the nuclei in the ROIs were manually marked using ImageJ software (Schindelin et al., 2012). We used the following criteria to exclude inappropriate nuclei from the analysis: Nuclei of glial cells that are much smaller and exhibited intensive staining with DAPI were excluded from the quantification (Chawla et al., 2004). Furthermore, broken and damaged nuclei or nuclei that were cut on the edge of the image plane were also excluded from the analysis. Cell assessments were performed manually in an experimenter-blind manner. A second experimenter verified the accuracy and reproducibility of the analysis by randomly selecting slices from the samples, doing manual cell counts, and then comparing their cell count findings with those of the other experimenter.

Table 1 summarizes the average number of nuclei (± standard deviation) analyzed for each image of each ROI (layer II, III, V-VI/IV-VI of middle and caudal areas 35 and 36). All analyzed nuclei that contained a nuclear Arc mRNA signal (Figure 1G, red dot indicated by white arrow) were identified as positive nuclei and manually counted. Then the percentage of Arc positive nuclei was calculated from all counted nuclei for each image and the average percentage of Arc mRNA positive nuclei from three brain sections of each animal was calculated for each of the ROIs. Finally, for each group, the mean percentage (± SEM) of Arc mRNA positive nuclei of each animal was calculated for each of the ROIs. Statistica software (TIBCO, CA, USA) was used for further analysis. The normal distribution of each data set was confirmed using the Kolmogorov-Smirnov test. A multifactorial analysis of variance (ANOVA) with four factors (group, area, compartments, and layer) and subsequent *post hoc* analysis (Fisher’s LSD test) was performed for statistical analysis to compare PRC areas 35 and 36 of the three groups (control, micro, or macro), layers (II, III, V-VI/IV-VI) as well as middle and caudal compartments. The level of significance was set to *p* < 0.05, and n corresponds to the number of animals (Table 1).

**Table 1:** Overview of the number of nuclei (mean ± standard deviation) analyzed for each image of each region of interest (ROI) for the layers of the middle and caudal PRC areas 35 and 36, as well as the number of animals (n) used in each group.

ROI			Average no. nuclei	Control (n)	Micro (n)	Macro (n)
area 35	middle	Layer II	88 ± 5.35	8	8	8
		Layer III	99 ± 5.77	8	8	8
		Layer V-VI	128 ± 2.70	8	8	8
	caudal	Layer II	86 ± 4.76	8	7	8
		Layer III	103 ± 5.30	8	7	8
		Layer V-VI	128 ± 3.38	8	7	8
area 36	middle	Layer II	96 ± 2.74	8	8	8
		Layer III	106 ± 3.61	8	8	8
		Layer IV-VI	123 ± 3.06	8	8	8
	caudal	Layer II	100 ± 5.00	8	7	7
		Layer III	104 ± 3.56	8	7	8
		Layer IV-VI	127 ± 5.01	8	7	8

Animal behavior (active exploration times and the number of rears) were assessed for each condition and statistically evaluated using one-way ANOVA (Table 2).

**Table 2:** Animal behavior in the microscale and macroscale cue paradigms.

A
**Exploration time (s)**	**Hole 1**	**Hole 2**	**Hole 3**	**Hole 4**	**one-way ANOVA**
	11.09 ± 1.58	12.88 ± 1.44	11.31 ± 1.61	12.31 ± 2.02	*F*_(3,60)_ = 0.2514 *p* = 0.860057
**Exploration time (s)**	**Object 1**	**Object 2**	**Object 2**		**one-way ANOVA**
	33.85 ± 5.17	45.00 ± 3.11	33.85 ± 4.06		*F*_(2,36)_ = 1.9840 *p* = 0.152276
B
	**Microscale items**	**Macroscale items**	**one-way ANOVA**
**Exploration time (s)**	276.8 ± 8.09	283.5 ± 10.32	*F*_(1,27)_ = 0.000 *p* = 0.987640
**No. rears**	21.07 ± 1.92	10.57 ± 0.88	*F*_(1,27)_ = 24.8538 ***p* = 0.000032**

*A. Exploration times at holes 1–4 of the holeboard (microscale item paradigm) that contained items in the holeboard holes, or of objects 1–3 of the macroscale item paradigm. One-way ANOVA revealed no significant difference in exploration times between either holes or objects*. *B. Total exploration time of environment and holes/objects in the microscale and microscale cue paradigms, and the number of rears conducted during exploration of the respective environments. Total exploration was not significantly different between the paradigms whereas animals exploring microscale items exhibited a significantly higher number of rears. Significant effects are highlighted in bold font.*

## Results

It is well described that the PRC is involved in the encoding of object recognition memory (Mumby and Pinel, 1994; Zhu et al., 1995a; Aggleton and Brown, 2005; Albasser et al., 2015). Some previous studies examined IEG expression separately for areas 35 and 36 (Albasser et al., 2010, 2013; Burke et al., 2012a). Nevertheless, these studies could not detect differences in the engagement of areas 35 and 36 in novel object learning, although the intrinsic connectivity of both areas, as well as several afferent connections, are distinct (Burwell and Amaral, 1998a, b; Burwell, 2000; Furtak et al., 2007). The present study aimed to examine the effect of novel learning about micro- and macroscale items in the layers (II, III, V-VI/IV-VI) of the middle and caudal areas 35 and 36. We assessed nuclear Arc mRNA expression in the region of interest in the PRC after novel (micro- or macroscale) item exploration.

Here, we verified that in all conditions where IEG expression was subsequently assessed, that adequate and equivalent object exploration occurred (Table 2). Thus, as reported previously (Sethumadhavan et al., 2020) animals paid equal attention to all of the objects placed in the holeboard holes in the microscale paradigm, and to all of the objects in the macroscale paradigm (Table 2A). The animals also spent almost all of the 5 assigned minutes actively exploring in the paradigms, whereby the degree of exploration was equivalent in the two paradigms (Table 2B). The number of rears was higher in the microscale, compared to the macroscale condition (Table 2B) condition. Effects were distinct from the control condition where animals simply remained stationary and at rest in one corner of the experimental chamber. Behavioral data were also reported in Sethumadhavan et al. (2020).

### Novel Object Learning Changes Nuclear Arc mRNA Expression in the Perirhinal Cortex

**Figure 2:**
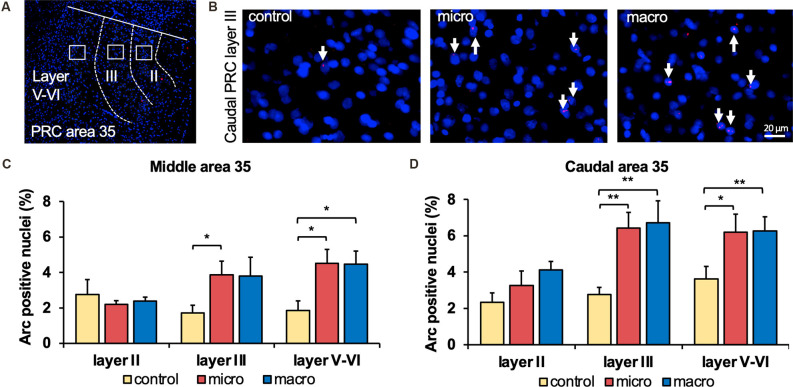
Exposure to novel microscale or macroscale items significantly enhances nuclear Arc mRNA expression in middle and caudal area 35. **(A)** Fluorescent image of area 35 showing the superficial layers II and III and the deep layers (separated by white dotted lines), as well as the origin of the representative photomicrographs (white outline). Nuclei were stained with DAPI (blue) and the Arc mRNA signal is shown in red. **(B)** Photomicrographs showing nuclear Arc mRNA expression (red, indicated by white arrows) in layer III of the caudal area 35 from a control animal (control) and an animal that participated in microscale (micro) or macroscale (macro) item exploration. Blue: nuclear staining with DAPI. Images were taken using a 20x objective. Scale bar: 20 μm. **(C)** The relative percentage of Arc mRNA positive nuclei in the middle area 35 of control and the two experimental groups (mean ± SEM) is shown. Novel exposure to microscale items (micro, red bar) triggers a significant increase in nuclear Arc mRNA expression in the superficial layer III and deeper cell layers of the middle area 35 compared to their control group (yellow bar). By contrast, novel exposure to macroscale items (macro, blue bar) significantly elevates nuclear Arc mRNA expression in the deep layers of the middle area 35 compared to control animals (Fisher’s LSD *post-hoc* test: *p* < 0.05), whereas a tendency is visible for layer III. No significant differences were observed in layer II of the middle area 35. **(D)** Relative percentage of Arc mRNA positive nuclei in the caudal area 35 of controls and the two experimental groups (mean ± SEM). The exposure to novel objects, regardless of the different dimensions of the novel items, significantly changes nuclear Arc mRNA in the superficial layer III and the deep layers of the caudal area 35 compared to their controls (*post-hoc* test). In layer II a tendency towards a change can be detected after the exploration of macroscale items (*post hoc*: caudal control vs. macro *p* = 0.077845). **(C,D)** Significant differences for each layer for the experimental groups compared to their control group are marked with asterisks *p < 0.05, **p < 0.01.

**Figure 3:**
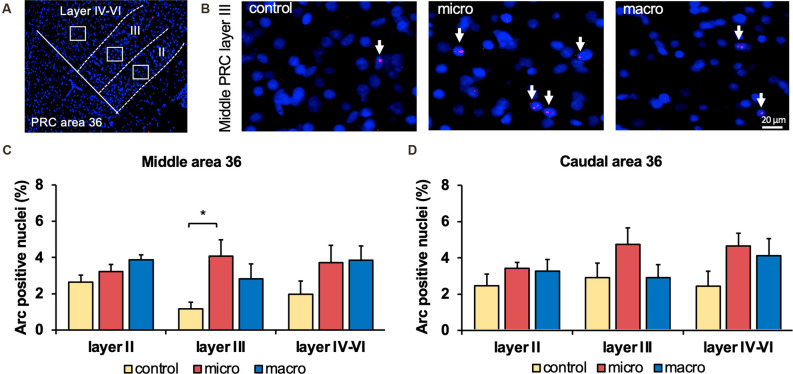
Neurons in layer III of area 36 respond to learning about microscale items. **(A)** Representative DAPI-stained coronal sections of the rat brain showing PRC area 36 and its layer organization (separated by white dotted lines). White squares indicate the origin of the representative photomicrographs. **(B)** Photomicrographs showing nuclear Arc mRNA expression (red, indicated by white arrows) in layer III of the middle area 36 of control animals (control) or animals that participated in microscale (micro) or macroscale (macro) item exploration. Blue: nuclear staining with DAPI. Images were taken using a 20x objective. Scale bar: 20 μm. **(C,D)** In the middle area 36 exposure to microscale items (micro) but not macroscale items (macro) triggers a significant increase in Arc mRNA expression in layer III compared to controls (*Post hoc* test: layer III: middle control vs. micro *p* < 0.05, caudal control vs. micro *p* = 0.079281). No changes in Arc mRNA expression were observed in layer II or layers IV-VI of the middle and caudal area 36. The relative percentage of Arc mRNA positive nuclei in the middle **(C)** and caudal **(D)** area 36 of the control and the two experimental groups (mean ± SEM). **(C,D)** Significant differences for each layer for the experimental groups compared to their control group are marked with an asterisk *p < 0.05.

Assessment of somatic Arc expression triggered by the behavioral paradigms used revealed that Arc mRNA expression differs significantly between the experimental groups (multifactorial ANOVA: *F*_(2,245)_ = 17.7158, *p* < 0.00001, Table 3). *Post hoc* analysis revealed that novel object exploration enhances nuclear Arc mRNA expression in both test groups (microscale (micro), macroscale (macro) items) compared to control animals (control: vs. micro *p* < 0.000001, vs. macro *p* < 0.00001), whereas no difference between the two test groups was detectable (micro vs. macro *p* = 0.714080). In general, Arc mRNA expression was different between areas 35 and 36 (multifactorial ANOVA: *F*_(1, 245)_ = 5.6150, *p* < 0.05), as well as the middle and caudal compartments (multifactorial ANOVA: *F*_(1,245)_ = 17.0604 *p* < 0.0001) and layers (multifactorial ANOVA: *F*_(2, 245)_ = 7.4685 *p* < 0.001) of the PRC (Table 3). Interestingly, a significant difference (independent of the experimental groups) was detected for PRC layers III and the deep layers compared to layer II (layer II: vs. III *p* < 0.01, vs. V-VI/IV-VI *p* < 0.001). By contrast, expression in layers III and V-VI/IV-VI was equivalent [layer III vs. (I)V-VI *p* = 0.382560]. This general comparison provided the first hint that micro- and macroscale item learning, but also layers III and V-VI/IV-VI may share similar mechanisms in enabling the encoding of object information. The interaction of all factors revealed no significant effect (Table 3, multifactorial ANOVA: *F*_(4,245)_ = 0.0933, *p* = 0.984504). Pairwise *post hoc* comparisons that provide deeper insights into the engagement of the layers and compartments of area 35 and 36 in micro- and macroscale object encoding are described in detail below. See also Figures 2, 3.

**Table 3:** Summary of the results of multifactorial ANOVA that was conducted for the factors: area (35,36), group (control, micro, macro), compartment (middle, caudal), and layer (II, III, V-VI/IV-VI).

Factor	ANOVA
compartment	*F*_(1,245)_ = 17.0604 *p* **< 0.0001**
area	*F*_(1,245)_ = 5.6150 *p* **< 0.05**
layer	*F*_(2,245)_ = 7.4685 *p* **< 0.001**
group	*F*_(2,245)_ = 17.7158 *p* **< 0.00001**
compartment*area	*F*_(1,245)_ = 7.3314 *p* **< 0.01**
compartment*layer	*F*_(2,245)_ = 2.2005 *p* = 0.112928
area*layer	*F*_(2,245)_ = 3.3488 *p* **< 0.05**
compartment*group	*F*_(2,245)_ = 0.1835 *p* = 0.832484
area*group	*F*_(2,245)_ = 1.5293 *p* = 0.218747
layer*group	*F*_(4,245)_ = 1.9075 *p* = 0.109785
compartment*area*layer	*F*_(2,245)_ = 0.4573 *p* = 0.633499
compartment*area*group	*F*_(2,245)_ = 1.2741 *p* = 0.281542
compartment*layer*group	*F*_(4,245)_ = 0.1844 *p* = 0.946349
area*layer*group	*F*_(4,245)_ = 0.9129 *p* = 0.457021
compartment*area*layer*group	*F*_(4,245)_ = 0.0933 *p* = 0.984504

Significant effects are highlighted in bold font.

### Novel Object Exposure Enhances Immediate Early Gene Expression in Perirhinal Cortex Area 35

**Figure 4:**
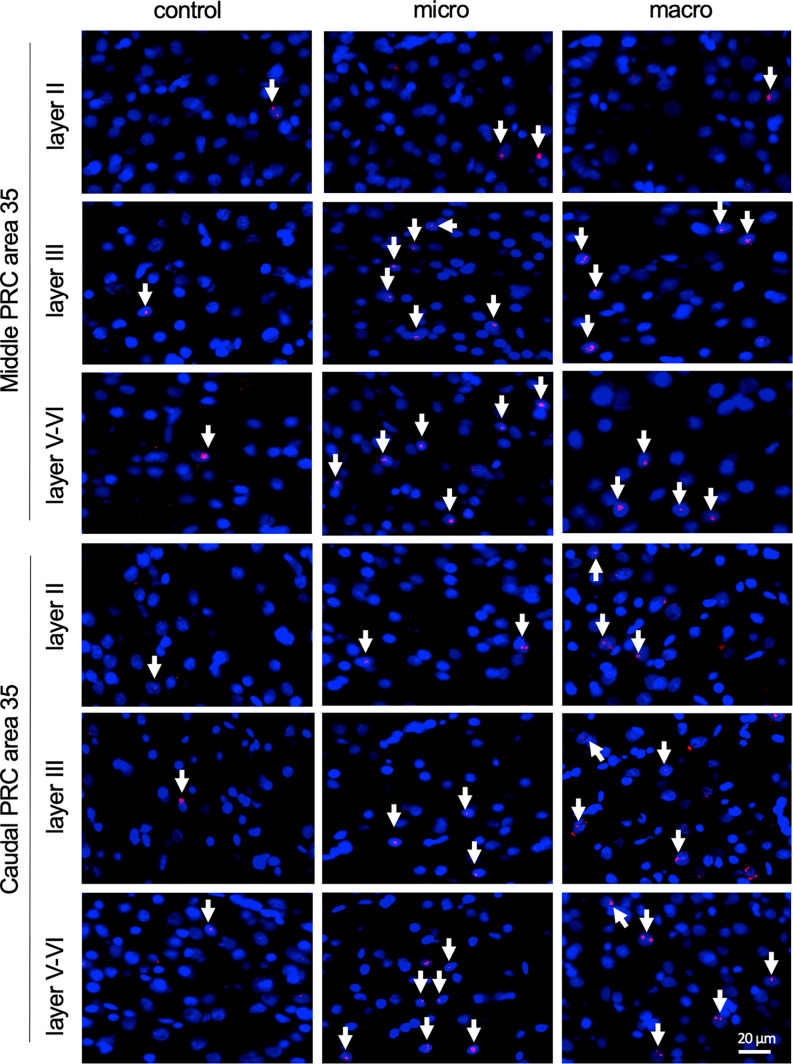
Nuclear Arc mRNA expression in the superficial and the deep layers of area 35 upon exposure to novel items. Photomicrographs showing nuclear Arc mRNA expression (red, indicated by white arrows) in layer II (top), III (middle), and the deep layers (bottom) of the middle and caudal area 35 of control animals (control) or animals that participated in microscale (micro) or macroscale (macro) item exploration. Nuclei (blue) are stained with DAPI. Images were taken using a 20x objective. Scale bar: 20 μm.

In the middle and caudal area 35, novel object learning resulted in a layer-specific change in nuclear Arc mRNA expression (Figures 2, 4). For layer II, learning about microscale items did not change IEG expression in the middle and caudal area 35 compared to control animals. Learning about macroscale items had no effect on Arc mRNA expression in layer II of the middle area 35, whereas the caudal area 35 exhibited a tendency towards a significant change in IEG expression in comparison to controls (*post hoc*: *p* = 0.077845, Table 4). These findings align to some extent with our previous report that macroscale item learning results in an increase in Arc mRNA expression in layer II of the caudal area 35 after macroscale item learning (Sethumadhavan et al., 2020). In the present study, a different imaging technique was used and a larger proportion of nuclei was analyzed in layer II (Figure 2, Table 1).

**Table 4:** The outcome of *post hoc* analyses (Fisher’s LSD test) to assess differences between the three groups (control, micro, and macro) of each layer of each compartment of areas 35 and 36.

			**Control vs. Micro**	**Control vs. Macro**	**Micro vs. Macro**
area 35	middle	Layer II	*p* = 0.594751	*p* = 0.726693	*p* = 0.855159
		Layer III	***p* < 0.05**	*p* = 0.050340	*p* = 0.934247
		Layer V-VI	***p* < 0.05**	***p* < 0.05**	*p* = 0.961146
	caudal	Layer II	*p* = 0.370561	*p* = 0.077845	*p* = 0.416642
		Layer III	***p* < 0.01**	***p* < 0.01**	*p* = 0.777222
		Layer V-VI	***p* < 0.05**	***p* < 0.01**	*p* = 0.950707
area 36	middle	Layer II	*p* = 0.551659	*p* = 0.213414	*p* = 0.515413
		Layer III	***p* < 0.05**	*p* = 0.327652	*p* = 0.168985
		Layer IV-VI	*p* = 0.141995	*p* = 0.108994	*p* = 0.892374
	caudal	Layer II	*p* = 0.351441	*p* = 0.433071	*p* = 0.885913
		Layer III	*p* = 0.079281	*p* = 0.999163	*p* = 0.079452
		Layer IV-VI	*p* = 0.160848	*p* = 0.357993	*p* = 0.605778

Significant effects are highlighted in bold font.

### Exploration of Micro- and Macroscale Objects Enhances Arc mRNA Expression in Layer III and Layers V-VI of Middle and Caudal Area 35

Analysis of layers III and V-VI of the middle and caudal area 35 revealed a significant increase in nuclear Arc mRNA expression upon exposure to objects of different dimensions in most of the layers (Figures 2, 4, Table 4). Exploration of macroscale items resulted in an enhanced expression of nuclear Arc mRNA in layer III of the caudal area 35 compared to controls (*post hoc*: control vs. macro *p* < 0.01). Layer III of the middle area 35 exhibited a tendency towards a significant change in IEG expression induced by learning about macroscale objects (*post hoc*: *p* = 0.050340, Table 4). Interestingly, layer V-VI of both compartments of area 35 exhibited a significant increase in nuclear Arc expression in animals that explored macroscale items in comparison to controls (*post hoc* middle control vs. macro *p* < 0.05, caudal control vs. macro *p* < 0.01), suggesting an engagement of layers III and V-VI of area 35 in the encoding of macroscale items. The exposure to microscale items resulted in a significant enhancement of nuclear Arc mRNA expression in layer III of the middle and caudal area 35 in comparison to their control groups (*post hoc*: middle control vs. micro *p* < 0.05, caudal control vs. micro *p* < 0.01). A similar change in IEG expression could be detected in layers V-VI after novel microscale item exploration compared to controls (*post hoc*: middle control vs. micro *p* < 0.05, caudal control vs. micro *p* < 0.05). These results indicate that the superficial cell layer III and the deeper cell layers of middle and caudal area 35 are likely to engage in novel micro- and macroscale item learning.

### Exploration of Microscale Objects Changes Arc mRNA Expression in Layer III of Area 36 of the Perirhinal Cortex

**Figure 5:**
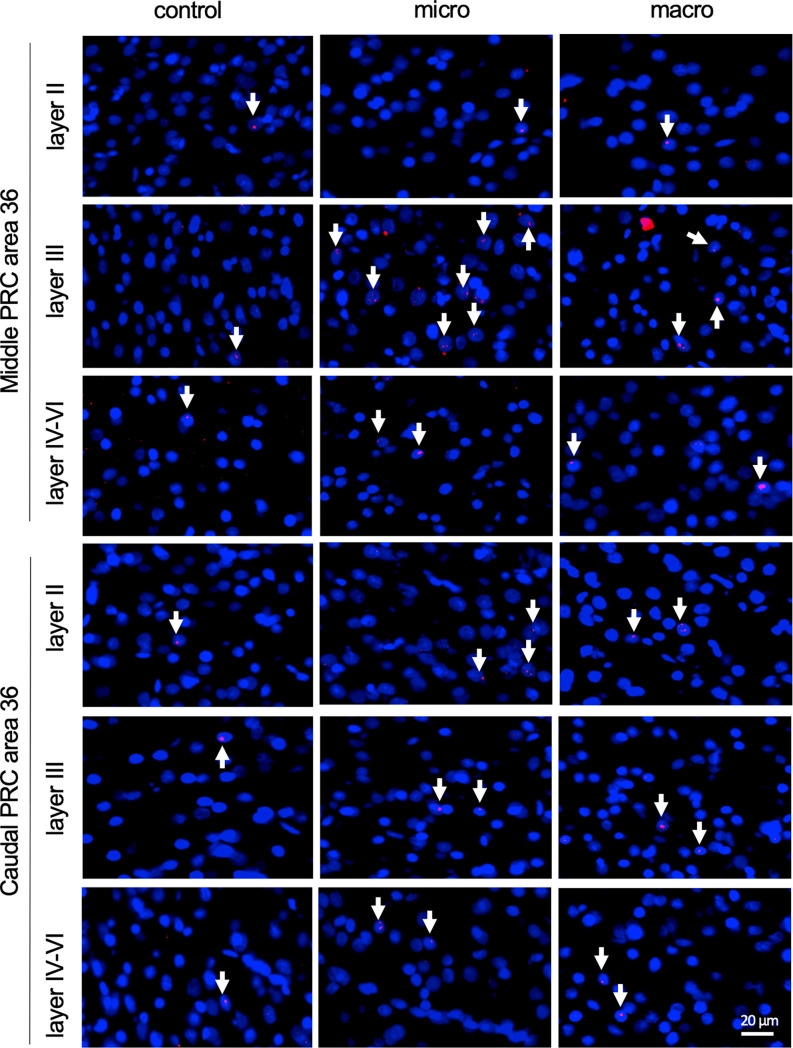
Nuclear Arc mRNA expression in the superficial and the deep layers of area 36 following exposure to novel items. Photomicrographs showing Arc mRNA expression (red, indicated by white arrows) in layer II (top row), III (middle row), and the deeper layers (bottom row) of the middle and caudal of area 36 from control animals (control) or animals that participated in microscale (micro) or macroscale (macro) item exploration. Nuclei (blue) are stained using DAPI. Images were taken using a 20x objective. Scale bar: 20 μm.

After detecting these general changes in Arc mRNA expression induced by novel micro- and macroscale item exposure in area 35, we examined the engagement of area 36 in the encoding of both types of items (Figures 3, 5, Table 4). Neither micro- nor macroscale items enhanced IEG expression in superficial layer II of the middle and caudal area 36 compared to controls (Figure 3, Table 4). Similarly, no difference in Arc mRNA expression was detected in the deep layers (IV-VI) of the middle and caudal area 36 upon presentation of either type of item (Figure 3, Table 4). In contrast, a distinct enhancement in nuclear Arc mRNA expression was induced by microscale item learning in layer III of the middle area 36 (Figure 3C, *post hoc*: control vs. micro *p* < 0.05), whereas macroscale item learning had no effect on IEG expression compared to the control group.

Examination of nuclear Arc mRNA expression in layer III of the caudal area 36 identified a tendency towards an increase induced by microscale item exploration in comparison to controls (Figure 3D, *p* = 0.079281). By contrast, exploration of macroscale items had no effect on IEG expression in layer III of the caudal area 36. Comparing nuclear Arc mRNA expression in layer III of the caudal area 36 in both test groups reveals another tendency towards a difference between the two paradigms (micro vs. macro *p* = 0.079452). These results suggest that superficial layer III of the middle and to some extent, caudal area 36, are involved in the encoding of microscale, but not macroscale, item information.

## Discussion

A role for the PRC in item recognition memory is well-described (Miranda and Bekinschtein, 2018). But recognition memory necessitates a comparison of the encountered item with a previously formed record. Furthermore, item discrimination is not only determined by item form, and features, but also item size. Here, we explored to what extent the PRC is involved in *de novo* encoding of item dimensions and further investigated to what extent the different cortical layers of areas 35 and 36 might be functionally differentiated in this regard. To this end, we examined somatic immediate early gene (IEG) expression in the superficial and deep layers of areas 35 and 36 induced by novel exposure to small and discrete (microscale) and large and overt (macroscale) objects. IEG expression triggered by these events was detected using cellular compartment analysis of temporal activity by fluorescence *in situ* hybridization. Overall, our results indicate that the different cortical layers of area 35 engage in *de novo* encoding of item identity, in a dimension-specific manner. Whereas layers III and V-VI of the middle and caudal area 35 engage in *de novo* encoding of both kinds of item identity, superficial layer II, plays a subordinate role in the novel encoding of only macroscale item information. By contrast, in area 36, layer III is specifically involved in the *de novo* encoding of microscale items. Taken together these data suggest that areas 35 and 36 play distinct roles in the encoding of novel information about item dimensions.

These differences may derive from the anatomical structure of areas 35 and 36. From a histological point of view, there are very distinct differences in the cortical layers between areas 35 and 36. Area 35 is considered to be an agranular cortex (lacking layer IV) whereas area 36 has a weakly pronounced layer IV (Burwell, 2001). In addition, area 35 exhibits no clear separation of layers II and III in comparison to area 36 (Burwell, 2001). Moreover, areas 35 and 36 can be further divided into dorsal and ventral subcompartments and area 36 has a third segregation into a posterior subcompartment (Burwell, 2001). In the present study, we did not further differentiate these subcompartments. Nuclear Arc mRNA expression was scrutinized from layers II, III, and (I)V-VI of the middle and caudal PRC, and thus, included nuclei from the entire dorsoventral axis of either area 35 or area 36.

The anatomical connections along the rostrocaudal axis of the PRC and between areas 35 and 36 also exhibit several interesting differences (Furtak et al., 2007): compared to all other PRC levels, caudal area 36 receives the strongest input from visual association areas, and also the input from the POR, which receives even stronger visual inputs, predominantly targets area 36 rather than area 35 (Burwell and Amaral, 1998a; Furtak et al., 2007). Thus, area 36 may be better equipped to process information about microscale items than area 35. In addition to strong inputs from visual association areas, area 36 receives stronger polymodal associational inputs compared to area 35 (Burwell and Amaral, 1998a), suggesting that highly pre-processed sensory information reaches area 36. Thus, microscale item processing by area 36 may be supported not only by visual, but also tactile and odor inputs, for example.

Scrutiny of the intrinsic connections of the PRC also reveals very distinct features of area 35 compared to 36: Area 36 strongly projects to all rostrocaudal levels and the projections may target preferentially more ventral levels within this area (Burwell and Amaral, 1998b; Burwell, 2000). In turn, mainly ventral area 36 projects to the same rostrocaudal level of area 35, whereas area 35 makes only weak intrinsic connections and provides only weak feedback projections to area 36 (Burwell and Amaral, 1998b; Burwell, 2000). These differences in intrinsic and reciprocal connectivity of area 35 and 36 could suggest that area 36 may forward visual object information to area 35.

It has been reported that the PRC supports spatial memory by intrinsic information encoding or support of pattern completion (Ramos and Vaquero, 2005; Ramos, 2008, 2017; Barry et al., 2016). For example, PRC lesions result in impaired retrieval of spatial memories (Ramos and Vaquero, 2005; Ramos, 2008). In addition, several weeks after novel spatial learning, enhanced expression of Arc and Fos proteins is evident in the PRC (Barry et al., 2016). This involvement in long-term encoding contrasts with studies that report no engagement of the PRC in the learning of novel spatial arrangements of familiar stimuli (Wan et al., 1999; Jenkins et al., 2004; Aggleton et al., 2012), but may reflect a temporal aspect to the engagement of the PRC in these processes. Given the timeline of our study, we assume that the response of the PRC to novel macroscale or microscale object presentation relates more to the presentation of the item themselves than their spatial configurations (Aggleton et al., 2012). But the differentiated responses of area 35 and 36 to item dimensions introduces the interesting possibility that the PRC may be able to discriminate item size.

If the PRC does not support item-place information encoding *per se*, the question arises as to how the hippocampus acquires key information to execute this task itself. Anterograde and retrograde tracing studies have reported an *indirect* connection from layers II/III of the areas 35 and 36 to the hippocampus *via* the LEC layer II/III, but projections of area 35 are more intense than of area 36 (Burwell and Amaral, 1998b; Pinto et al., 2006; Doan et al., 2019). Moreover, electrical stimulation of layer II/III of area 35 results in postsynaptic potential in the neighboring LEC, supporting monosynaptic inputs to layer II/III of LEC originating mostly from layer II/III of area 35 (Doan et al., 2019). These are likely to play a key role in the processing of novel object memory. Consistent with these anatomical observations, layer III of both middle and caudal area 35 exhibits an increase in activity as a consequence of both microscale and macroscale item encoding. This result indicates that neurons in this layer of area 35 process novel object information independently of the dimensions of the objects.

The hippocampal CA1 region plays an important role in the learning about microscale item-place configurations (Kemp and Manahan-Vaughan, 2004; Hagena and Manahan-Vaughan, 2011; Hoang et al., 2018), and the microscale and macroscale behavioral paradigms used in the current study trigger both synaptic plasticity and somatic IEG expression in distinct subcompartments of the hippocampus (Hoang et al., 2018; Hoang and Manahan-Vaughan, 2021). Interestingly, it has been reported that a *direct* projection from neurons in the superficial layers of the PRC to the CA1 region and more strongly to the subiculum of the hippocampus exists (Naber et al., 1999; Furtak et al., 2007; Agster and Burwell, 2013; Suter et al., 2013). In our study, we observed that layer III of area 36 was activated only by the exposure to novel microscale items, and unaffected by macroscale items. This finding prompts the possibility that the engagement of layer III of area 36 in microscale object encoding and the weak *direct* connection from the PRC to the hippocampus (Furtak et al., 2007; Agster and Burwell, 2013) may support microscale item-place encoding in the hippocampal CA1 region.

The hippocampal dentate gyrus region engages in macroscale item-place encoding (Kemp and Manahan-Vaughan, 2008; Hoang et al., 2018, 2021). This prompts the question as to whether this process may be supported by macroscale item encoding in area 35. In contrast to the direct connections of PRC with the CA1 region, tracing studies have generated conflicting results with regard to a possible *direct* connection from the PRC to the dentate gyrus: Several anatomical studies indicate that the dentate gyrus does not receive projections from the PRC (Kosel et al., 1983; Mcintyre et al., 1996; Naber et al., 1999), whereas others argue that the PRC/entorhinal cortex inputs terminate on in the middle third of the molecular layer of the dentate gyrus, which can be correlated to the medial perforant path input (Canning and Leung, 1997; Canning et al., 2000). However, a more recent study suggests that a monosynaptic input extends from the superficial layers of the PRC at least to newborn granule cells (Vivar et al., 2012). Lesioning this particular pathway results in impaired pattern separation (Vivar et al., 2012), indicating that an interconnection between the DG and the PRC may exist. In the current study, neurons in layer II of the PRC exhibited only sparse activity in response to novel object learning. These neurons, in particular in the caudal compartments of area 35, only become active when animals engage in the exploration of novel macroscale, but not microscale items. These results suggest in particular that activity in layer III of area 36 and to a smaller extent also layer II of area 35 may be input-specific. Keeping in mind that the PRC mainly indirectly projects to the hippocampal formation, it is likely that activation of the superficial layers II and III may be correlated with an encoding of novel object information that is transmitted *via* the LEC to the hippocampus. Here, information about novel objects might be projected from PRC superficial layers to the LEC that in turn sends information either directly or indirectly to the related hippocampal compartments for the encoding of the spatial components of the object configuration.

**Figure 6:**
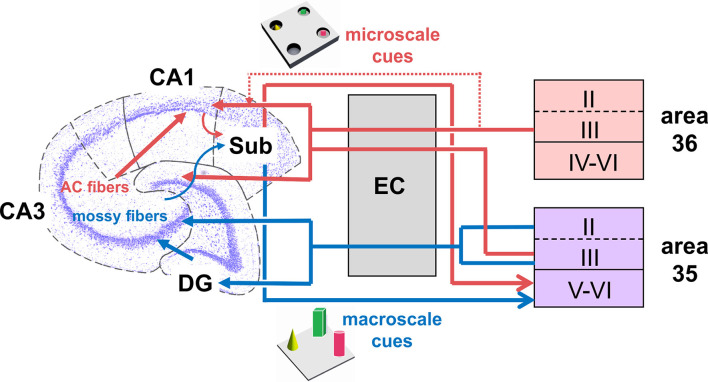
Hypothesis of projections within hippocampal-perirhinal cortex circuits in novel object learning. Novel acquisition of item information (macroscale, blue) activates neuronal encoding in superficial and deep layers in area 35. Information about novel macroscale items may enter the entorhinal cortex through layers II and III of mostly area 35 before it reaches the hippocampus *via* the perforant path (Witter et al., 2000; Doan et al., 2019). In the hippocampus, the dentate gyrus and mossy fibers-CA3 synapses process this information (Kemp and Manahan-Vaughan, 2008; Hagena and Manahan-Vaughan, 2011; Hoang et al., 2018). The processed information may then be transferred to the subiculum, which presumably, in turn, sends information back to the deep layers of area 35 (Swanson and Cowan, 1977; Deacon et al., 1983; van Groen and Wyss, 1990; Kloosterman et al., 2003; Agster and Burwell, 2013). According to our results, area 36 does not support the encoding of this kind of information. If the item dimensions are small (microscale items, red), the Schaffer-collateral-CA1 and commissural-associational (AC)-CA3 synapses process this information (Kemp and Manahan-Vaughan, 2008; Hagena and Manahan-Vaughan, 2011; Hoang et al., 2018). Acquisition of microscale items (red) enhances neuronal activity in the superficial layer III of areas 35 and 36. This information may then be sent to the entorhinal cortex which in turn forwards the information to the Schaffer-collaterals-CA1 and AC-CA3 synapses in the hippocampus. In addition, information directly originating from the PRC superficial layers may be of particular importance for microscale information processing in CA1/subiculum (Agster and Burwell, 2013). Finally, the subiculum receives microscale item information that may be sent back to the deep layers of area 35 (Swanson and Cowan, 1977; Deacon et al., 1983; van Groen and Wyss, 1990; Kloosterman et al., 2003; Agster and Burwell, 2013).

In our study, neurons in the layers V-VI of area 35 encoded both kinds of items presented, suggesting that item dimension is not discriminated in these layers. It has been reported that the back projections from the CA1 region/subiculum preferentially target the layers V-VI of the caudal PRC (Swanson and Cowan, 1977; Deacon et al., 1983; van Groen and Wyss, 1990; Kloosterman et al., 2003). Thus, another interesting possibility is that the hippocampus may send general information about object dimensions and/or item-place both directly and indirectly *via* the LEC back to the PRC (Burwell and Amaral, 1998b). This information may then be encoded in layer V-VI of area 35 (see: Figure 6). In order to find out if activity in these layers may reflect novel encoding or recognition/retrieval (Burke et al., 2012a), and/or spatial components of object configurations (Ramos and Vaquero, 2005; Ramos, 2008), further investigations will be necessary.

Our study indicates that areas 35 and 36 are functionally differentiated with regard to their role in novel item dimension encoding. Differences in the anatomical connections of areas 35 and 36, and differences along their rostrocaudal levels indicate that these structures are also functionally differentiated with regard to object recognition memory (Burwell and Amaral, 1998a, b; Furtak et al., 2007; Agster and Burwell, 2013). Empirical evidence for this, in particular in rodents, is sparse but findings indicate a differentiated involvement of areas 35 and 36 in both object recognition memory and novel item encoding (Fujimichi et al., 2010; Sethumadhavan et al., 2020). Most of the studies that examined the PRC used substance injections or lesions of the PRC, to understand the role of the PRC in object recognition memory (Mumby and Pinel, 1994; Winters and Bussey, 2005; Albasser et al., 2009; Aggleton et al., 2010). These methods, although useful in gaining an understanding of the role of PRC in object recognition and related behavioral tasks, offer limited potential for the functional differentiation of area 35 and area 36.

The IEGs, Arc and cfos, can be used as biomarkers of neuronal activity and synaptic plasticity (Zhu et al., 1995b; Guzowski et al., 1999, 2000; Burke et al., 2012a). Most studies examined Fos expression in the PRC to determine the regional distribution of activated neuronal populations in conjunction with object recognition (Zhu et al., 1995b; Albasser et al., 2010, 2013; Seoane et al., 2012). Novel object learning in darkness increases Fos protein levels in the rostral areas 35 and 36, indicating its role in darkness perception (Albasser et al., 2013). In contrast, novel object learning in illuminated conditions enhances Fos expression in the caudal areas 35 and 36 (Zhu et al., 1995b; Albasser et al., 2009, 2010, 2013). Interestingly areas 35 and 36 of the middle compartment of the PRC exhibit similar levels of Fos protein for the novel object exposure and object recognition groups (Albasser et al., 2010, 2013). However, none of these studies distinguished Fos expression between the layers of the PRC (Zhu et al., 1995b; Albasser et al., 2010, 2013; Seoane et al., 2012). The expression of Fos protein peaks approximately 90–120 min after the start of an experience (Kovacs, 1998; Zangenehpour and Chaudhuri, 2002). This imprecise onset of transcriptional activation of the Fos protein results in a low temporal resolution of Fos protein expression, which can also confound interpretations of IEG expression related to a learning event.

In contrast to the Fos protein, the activation of Arc gene expression by a learning event is rapid and dynamic with the peak expression of Arc mRNA in cell nuclei occurring within 5–6 min of commencing an exploration period (Guzowski et al., 1999; Guzowski, 2002). This approach has the advantage of excluding that events that occurred before or after the exploration period do not inadvertently affect gene expression. This, in turn, allows a more precise mapping of activated neurons using Arc mRNA compared to Fos protein. Only a few studies have used somatic Arc expression to scrutinize the role of the PRC in object memory. Nonetheless, blocking Arc expression in the PRC of rodents, leads to an impairment in the differentiation of similar object representations, without affecting spatial, or distinct object representations (Miranda et al., 2017). These findings align with studies supporting the role of the PRC in resolving feature ambiguity (Bartko et al., 2007). Another study of Arc mRNA expression in layer V of areas 35 and 36 (Burke et al., 2012a), compared the effects of a novel context using the same object constellation twice (Burke et al., 2012a). The results indicated that novel and familiar exploration of objects results in enhanced Arc mRNA expression in layer V (Burke et al., 2012a), but interestingly a difference in Arc expression between areas 35 and 36 was not detected. In the present study, we detected a differentiated engagement of areas 35 and 36 in novel object learning, suggesting an area and layer specific activity of the PRC in novel object encoding that is related to item dimensions.

Comparing our behavioral task with the behavioral approach used by others reveals that most groups presented large-scale objects that were placed on the floor of an arena (Winters et al., 2004; Balderas et al., 2008; Albasser et al., 2009, 2010; Aggleton et al., 2010), that in many respects reflect our use of macroscale items. In some other studies, objects only could be explored visually through holes because they were presented indirectly behind one-way mirrors in paired-viewing tests (Zhu et al., 1995a,b, 1996), similar to our microscale approach where small items were placed in holes on the floor and could only be seen if the animals poked their noses into the holes. Some of these studies used objects with different dimensions (largest dimension: 3–15 cm; Zhu et al., 1995b) or height (5–20 cm; Winters et al., 2004), but the behavioral and neuronal response to different scales of objects was not explicitly examined (Zhu et al., 1995a,b; Winters et al., 2004). Interestingly in “paired-viewing” studies, it was reported that the use of “2D” pictures that were passively viewed on a screen instead of “3D” objects that were actively explored, generates a similar PRC encoding response: enhanced Fos levels can be detected in the PRC only for single novel pictures (not constellations) or novel objects compared to the re-exposure to either condition (Zhu et al., 1995b, 1996; Wan et al., 1999; Seoane et al., 2012).

It is important to emphasize that our study explored IEG expression triggered by novel object exposure. Most other studies compared IEG expression between a novel exposure and re-exposure (item recognition) state (Albasser et al., 2010, 2013) in the absence of a naïve control. The difference in our findings compared to the findings of these studies may thus serve to highlight how different compartments of the PRC support novel item processing, as opposed to item recognition. Along these lines, it is possible that there is no clear border for an involvement of the middle, and caudal compartment in novel visual object recognition memory, rather a graded change may occur from anterior to posterior in the strength of involvement of the PRC in this process. By contrast, a differentiated involvement of areas 35 and 36 in novel item memory is evident that is based on item dimensions.

The duration of exposure to the items may also play a role, however. We presented a constellation of three different objects for a duration of 5 min. Burke and colleagues (Burke et al., 2012a) also used a 5-min exploration period, but in contrast to our study, they presented five different objects. Due to a decrease in exploration time in the second exposure, they concluded that the animals remembered the objects presented (Burke et al., 2012a). A study using the same behavioral tasks that we used revealed a longer exploration time of the novel compared to the familiar object, confirming that the animals remembered the previously presented objects (Hoang et al., 2018). The difference in the number of objects is probably not the reason why we detected no change in IEG expression in area 36 in layer IV-VI in response to novel object recognition, in contrast to the similar response of layer V of areas 35 and 36 reported by Burke et al. (2012a). It is more likely that the results differ because we not only analyzed layer V but also included nuclei of layers (I)V-VI in our analysis.

## Conclusion

Taken together, the results from our current study suggest a layer- and area-specific involvement of the PRC in the processing and encoding of information about novel large- and small-scale objects. Whereas area 35 exhibits an increase in neuronal activity induced by object learning that is generally independent of object dimension, area 36 only engages when animals explore less prominent microscale items. Furthermore, differences in object dimension trigger layer-specific gene encoding in the PRC: neurons in layer III of area 36 are only involved in learning about microscale items, suggesting that distinct afferent and efferent connections may be responsible for the integration of less prominent item information. By contrast, the superficial layer III together with layers V-VI of area 35 are activated by both types of object dimensions. In conclusion, these findings suggest that areas 35 and 36 are functionally specialized to enable item encoding on the basis of item dimensions.

## Data Availability Statement

The raw data supporting the conclusions of this article will be made available by the authors, upon reasonable request.

## Ethics Statement

The animal study was reviewed and approved by Landesamt für Arbeitsschutz, Naturschutz, Umweltschutz und Verbraucherschutz, Nordrhein Westfalen.

## Author Contributions

DM-V and CS devised the concept and experimental strategy of the study. Experiments were conducted by NS and CS. Data analysis was conducted by NS, CS, and T-HH. DM-V wrote the article, with contributions from all authors. All authors approved the submitted version.

## Conflict of Interest

The authors declare that the research was conducted in the absence of any commercial or financial relationships that could be construed as a potential conflict of interest.

## Publisher’s Note

All claims expressed in this article are solely those of the authors and do not necessarily represent those of their affiliated organizations, or those of the publisher, the editors and the reviewers. Any product that may be evaluated in this article, or claim that may be made by its manufacturer, is not guaranteed or endorsed by the publisher.
